# Why doctors are not satisfied with their job-current status in tertiary care hospitals

**DOI:** 10.12669/pjms.35.1.72

**Published:** 2019

**Authors:** Faiza Sadaqat Ali, Bader Faiyaz Zuberi, Tazeen Rasheed, Majid Ahmed Shaikh

**Affiliations:** 1 Faiza Sadaqat Ali, FCPS. Civil Hospital, Karachi, Pakistan; 2 Bader Faiyaz Zuberi, FCPS. Dow Medical College, Dow University of Health Sciences, Karachi, Pakistan; 3 Tazeen Rasheed, FCPS. FCPS. Dow Medical College, Dow University of Health Sciences, Karachi, Pakistan; 4 Majid Ahmed Shaikh, FCPS. Dow Medical College, Dow University of Health Sciences, Karachi, Pakistan

**Keywords:** Job satisfaction, Burn out

## Abstract

**Objective:**

To determine level and factors of job satisfaction among doctors working in tertiary care hospitals in Pakistan.

**Methods:**

This is a multi-center cross-sectional survey conducted among Post graduate trainees, medical officers, consultants and faculty doctors. Job satisfaction was measured using 35 specific questions about sources of work-related stress and sources of work-related satisfaction. Satisfaction was defined if mean score of a factor was≥3.0, where factors were rated using a 5-point Linkert scale ranging from 1 (completely dissatisfied) to 5 (completely satisfied).

**Results:**

In this study 373 doctors participated, out of which 215(57.6%) were males. Over all mean satisfaction score was of 2.69 ±0.37. Departmental mean satisfaction scores were Internal medicine 2.71 ±0.35, Medical subspecialties 2.63 ±0.38, Surgical and allied 2.73 ±0.45. Designation means were Consultant 2.87 ±0.38, Faculty 2.78 ±0.44, Medical officer/ Registrar 2.50 ±0.32, Post graduate trainee 2.71 ±0.45. Public and private sector means satisfaction scores were 2.53 ±0.80 and 2.92 ±0.84 respectively.

**Conclusion:**

Job dissatisfaction was seen among doctors from all the tiers and departments. Public sector doctors were more dissatisfied than private sector doctors. Increasing age, duration of current posting and working experience, positively correlated with satisfaction level.

## INTRODUCTION

Medicine has always been considered as a sacred profession. In Pakistan too, many children dream to become doctor since their early childhood.^1^ On the one hand practicing medicine is highly satisfying and fulfilling while on the other hand this field is very demanding and stressful. Gradually a physician’s job has lost its charm as it used to be in past. According to the World Health Organization health care labor shortage is increasing globally especially where healthcare performance indicators are the not good.^2^ WHO in its report during the Third Global Forum on Human Resources for Health published the fact that by 2035 the world will be short of 12.9 million healthcare workers.^2^ Job satisfaction is the difference between a person’s expectation and his reward. If the summation of these influences gives rise to feelings of satisfaction, the individual has job satisfaction.^3^

There are two dimensions to job satisfaction

- Intrinsic-motivation, for example responsibility and recognition, which arises from within and does not require any external reward, which enable higher satisfaction and performance and
- Extrinsic-motivation like job security, salary, bonuses and working conditions etc. that keep maintained that performance even when the work is no longer remain pleasurable. The absence of extrinsic factors helps diminishing dissatisfaction.^4^

Satisfaction at job is vital to attain superlative quality of work.^5^,^6^ A physician’s satisfaction from his job considerably enhances his services and proportionately affects level of patient’s satisfaction with delivery of health care.^6^-^8^ The way health care facilities are being delivered has changed considerably over the last decade^9^ and doctors are no longer held in the high regard as in the past.^10^ Among doctors is Professional burnout is common among training and practicing physicians.^11^-^14^ Lack of autonomy, ability to provide high-quality patient care and freedom to make clinical decisions, has been reported as the most consistent deterrent to physician satisfaction and cause of burnout.^15^ It has been seen that the doctors who are not satisfied with their jobs have less satisfied patients and are more prone to suffer from physical and mental illness.^16^,^17^ Dissatisfied physicians may also deter future students from entering the field of medicine.^17^ This lack of satisfaction among doctors lead to migration to more privileged countries, which causes net shortage of competent doctors with mass destruction of health care system.

Job satisfaction surveys among doctors have been carried out in some parts of the country, using qualitative methods supplemented by quantitative methods.^18^ But these studies had small sample sizes, single centered and lack of anonymization.^19^,^20^ Multicenter large studies in which data collected anonymously was needed to study job satisfaction properly. Present study was conducted with the objectives to determine the level of job satisfaction in doctors working in different tertiary care hospital within Pakistan anonymously to find out the factors associated with the lack of their job satisfaction, which if timely managed, can sustain and improve the overall efficiency, effectiveness and productivity of health care systems.

## METHODS

An online close-ended survey was conducted among doctors posted in different tertiary care hospitals within Pakistan during April 2018 to July 2018. Survey was conducted anonymously, and no personal data was collected. Post graduate (PG) trainees, Medical officers (MO)/Registrar, consultants and faculty members from different departments of tertiary care teaching hospitals within Pakistan who were working for at least three months in their current posting were included in the study. Those who refused to consent were excluded from the study.

Data was collected using an online, self-administered, close ended questionnaire consisting of demographic attributes of participants and domains and factors of job satisfaction. The job satisfaction questionnaire used was specifically developed & validated by Kumar and Khan, aimed to measure the job satisfaction among medical personnel.^21^ It consists of 35 factors addressing the seven domains of job satisfaction.

1. Satisfaction regarding salary and designation
2. Interpersonal relation and cooperation
3. Working environment
4. Patient relationship
5. Organization facilities
6. Career development
7. Human resource issues.

All factors were rated using a 5-point Linkert scale ranging from 1 (completely dissatisfied) to 5 (completely satisfied), the higher values indicated higher level of satisfaction. On-line survey form was created and hosted on internet using Google Forms. Invitation for participation in survey was dispersed using email and WhatsApp. Data was stored encrypted and password protected on Google Drive automatically as participant responded, with access only with first author. After completion of study the survey was closed, and data imported in SPSS for analysis. SPSS software version 23.0 was used for statistical analysis.

Using the reported frequency of job satisfaction of 59.6%,^22^ power of 95% and alpha of 0.05, the sample size was estimated of 347. PASS version 16 software was used for sample size calculation by testing One Proportion using the two-sided Z-Test with S(P0). Satisfaction status with the current job was the outcome variable. Satisfaction was defined if mean score of a factor was ≥ 3.0. Overall job satisfaction was calculated with regards to different factors within the domains. Background predictor variables were age, gender, department, designation, sector, duration of current posting and total duration of working experience. Continuous data was expressed as mean±standard deviation (SD) and was analyzed using Student’s t-test or ANOVA where applicable. Categorical data was analyzed via χ^2^- test. P-value of ≤ 0.05 was considered as significant. Pearson’s correlation was applied on age, duration of current posting & total duration of working experience with satisfaction score.

## RESULTS

In our study 373 individuals participated in the survey. Mean age ±SD of male and female participants were 37.2 ±9.9 and 43.2 ±6.0 years respectively. Mean duration at current posting for male and female participants were 4.7 ±5.0 years and 2.8 ±2.8 years respectively. Mean total working experiences of male and female participants were 10.6 ±9.3 and 5.4 ±4.7 years respectively. Rest of the demographic variable with their frequencies and mean satisfaction scores are mentioned in Table-I.

**Table-I:** Demographic variables, their frequencies and mean satisfaction scores.

	Variables	n (%)	Mean Satisfaction	SD
Gender	Male	215 (57.6)	2.66	0.86
	Female	158(42.4)	2.74	0.82
Sector	Public	219(58.7)	2.53	0.80
	Private	154(41.3)	2.93	0.84
Department	Internal Medicine	190(50.9)	2.71	0.86
	Medicine & Allied	120(32.2)	2.63	0.82
	Surgical & Allied	63(16.9)	2.73	0.83
Designation	Consultant	57(15.3)	2.86	0.93
	Faculty	68(18.2)	2.77	0.81
	MO/Registrar	94(25.2)	2.50	0.79
	PG Trainee	154(41.3)	2.71	0.82

The internal reliability of our survey was tested using Cronbach’s Alpha and was found as 0.95. Score of ≥ 0.7 is considered reliable. The cut off value for satisfaction was set at 3.0. In our study participants were found to be overall dissatisfied with their jobs. The mean total satisfaction score in our study was of 2.69 ±0.37. Satisfaction according to domains is mentioned in Table-II.

**Table-II:** The mean satisfaction scores ±SD according to domains.

Domains	Mean Satisfaction Score	SD
Privileges attached with job	2.32	0.87
Interpersonal relation and cooperation	2.93	1.01
Working environment	2.88	0.98
Patient relationship	2.76	1.07
Organization facilities	2.86	1.18
Career development	2.61	1.01
Human resource issues	2.53	0.10

Job satisfaction scores were correlated with age, duration of current posting and total duration of working experience. We found positive correlation of job satisfaction with increasing age (p = 0.006), duration of current posting (p= 0.003) and total duration of working experience (p= 0.043).

Although overall job satisfaction scores were below satisfaction level, but some of the factors within domains had satisfactory scores, these included current designation (3.28 ±1.23), location of health facility (3.21 ±1.34), retirement age (3.18 ±1.28), respect from subordinates (3.16 ±1.11), facility of electricity (3.08 ±1.51), supervision and support by senior (3.03 ±1.20), behavior of patient (3.03 ±1.10) and cooling facility in summer (3.02 ±1.56). Among these overall job satisfaction factors, the highest overall job satisfaction was present for individuals’ current designation. Remaining factors like children education allowance, home allowance, salary, working environment, work load, working hours, drinking water facility, job security, family and work balance were found to be dissatisfying, least satisfaction was seen for children education allowance.

Although doctors from all tiers were over all dissatisfied, but medical officer/registrar were more markedly dissatisfied as shown in Table-I. Consultant and faculty doctors both showed satisfaction with interpersonal relationship with means ±SD of 3.20 ±1.05 and 3.15 ±0.96 respectively. Both tiers were also satisfied with working environment with means ±SD of 3.00 ±1.04 and 3.06 ±0.96. Consultant doctors were also satisfied with organization facilities and patient relationship with means ±SD of 3.12 ±1.21 and 3.00 ±1.14 respectively. No difference in satisfaction level was found based on gender or department as all were dissatisfied overall. Detail are shown in Table-1.

Participants job satisfaction with regards to public and private sectors revealed that both sectors were dissatisfied with salary and other privileges of job, career development and human resource issues. While for organization facilities, working environment, interpersonal and patient relationship, private sector doctors were satisfied but public sector doctors were not satisfied. Details are shown in Table-III.

**Table-III:** Job satisfaction among public and private sector doctors.

Domains of job satisfaction	Sector	N	Mean	SD	SE Mean	P-value
Privileges of Job	Public Sector	219	2.25	0.83	.05638	0.065
	Private Sector	154	2.42	0.92	.07396	
Interpersonal Relations	Public Sector	219	2.86	1.00	.06783	0.124
	Private Sector	154	3.03	1.02	.08217	
Working Environment	Public Sector	219	2.77	0.95	.06414	0.014*
	Private Sector	154	3.02	1.01	.08100	
Patient Relationship	Public Sector	219	2.43	0.99	.06725	0.000*
	Private Sector	154	3.23	0.99	.07995	
Organization Facility	Public Sector	219	2.44	1.02	.06897	0.000*
	Private Sector	154	3.45	1.13	.09136	
Career Development	Public Sector	219	2.47	1.02	.06879	0.002*
	Private Sector	154	2.80	0.98	.07910	
Human Resource Issue	Public Sector	219	2.45	0.96	.06498	0.056
	Private Sector	154	2.65	1.02	.08254	

* Significance level P ≤0.05

## DISCUSSION

In our study majority of the participants were not satisfied with their job. Over all most of the dissatisfaction was found with children education allowance, home allowance, salary, working environment, work load, working hours, drinking water facility, job security and family and work balance. In contrast, overall satisfaction was observed with current designation, location of health facility, retirement age, respect from subordinates, facility of electricity, supervision and support by senior and behavior of patient.

In Pakistan health care facilities are provided by public and private sector hospitals. In our study private sector doctors showed satisfaction with organization facilities (cooling facility in summer, drinking water and electricity facility, location of health care facility etc.), patient relationship (behavior of patients, quality of care, supply of essential items and implementation of health programs), interpersonal relationship (support and appreciation from boss and seniors, respect from juniors and discipline), working environment and provision of training, but showed lack of satisfaction with privileges attached with jobs (Salary, designation, children education, accommodation and conveyance allowances), HR issues (work load, working hours, family and work balance etc.) and career development. Public sector doctors who are providing services to a major bulk of poor patients showed satisfaction only with their designation, respect from their subordinates and retirement age. Rest of the factors like facilities provided by the organization, working environment, privileges attached with jobs, quality of health care facilities, career development, recruitment process, work load and balance between work and family were dissatisfactory for them. In our study all tiers of doctors from postgraduate trainee, medical officer/registrar, consultant and faculty found to be overall dissatisfied with their job. Gender and departments did not constitute any significance difference in satisfaction level as all were dissatisfied.

Main reason of this very high job dissatisfaction in public sector is that they are under resourced, as Government spending only 0.5% of GDP or US$ 6.4 per capita on health which is apparently not enough to keep the morale high for doctors.^23^ A recent study conducted in Government hospital of eastern India reported that more than half (59.6%) doctors were satisfied with their job and the most important factor was found to be working space. This is in contrast with our study, where majority are not satisfied and lack of facilities at working space, salary and career growth are the main concerns. Tasneem S et al studied job satisfaction in a public tertiary care teaching hospital in Rawalpindi, Pakistan^19^ on 89 health related professionals, showed that greater satisfaction with their supervisors, responsibilities, nature of job as well as relationship with their colleagues but showed lack of satisfaction for the remaining factors like salaries, additional benefits, communication and working conditions.^19^ Atif K et al in his survey among 97 doctors working in tertiary care hospital in Lahore showed that only 13.3% participants enjoyed their job with high satisfaction.^20^ They also reported the poor job satisfaction in relation to age, service years, education and salary,^20^ possibly because of the same government lack of proper budget allocation to health care system. These studies had limitation of being single centered and small sample size, assessed few modifiable factors and did not assess private sector doctor’s satisfaction level. People do not respond favorably to restrictive work environments, therefore, it becomes essential for organizations to create incentivized environment for the employees to achieve the highest level of job satisfaction.^24^

In our study we assessed most of the modifiable factors that could impact the level of overall job satisfaction among doctors not only in public but also in private sector. We used a reliable tool for data collection and since it was an online survey so no error in obtaining and filling the survey Form.

### Limitations of the study

It is a cross-sectional study design as no temporality can be assessed. Since all measures were self-reported; limitations in subjective understanding and comprehension could not be denied, it is therefore possible that the respondents might have over or under-reported their level of satisfaction. Generalization of research could not be guaranteed. By highlighting this fall in job satisfaction among doctors we want to sensitize the higher authorities to make valuable amendments in health care policies so that the “curers” and the “healers” will find no reason to leave their own country.

## CONCLUSION

Fall in job satisfaction was seen among doctors from all the tiers and disciplines. Public sector doctors were more dissatisfied than private sector doctors. In public sector organization working facilities, working environment, salary, allowances, career development and work load were main concerns. While in private sector, privileges attached with jobs like children education, accommodation and conveyance allowances, workload, working hours, family and work balance, career growth and promotions were the main problems causing dissatisfaction at their job.

### Authors’ contributions

**FSA:** Prepared manuscript, collected data, gave final approval of manuscript.

**BFZ:** Designed and conceived the study, did statistical analysis and final approval of study.

**TR & MAS:** Collected data, literature search and initial draft write up.
